# Supplementary material for: MetaRibo-Seq measures translation in microbiomes
Source: Nat Commun. 2020 Jun 29;11:3268. doi: 10.1038/s41467-020-17081-z (PMC7324362; doi:10.1038/s41467-020-17081-z)
Supplement: Supplementary file 10 — Supplementary Data 7 [file 41467_2020_17081_MOESM10_ESM.zip › File2/Confidence_High_Taxonomy/220638_out.krona.html]

Javascript must be enabled to view this page.

members
magnitude
magnitudeUnassigned
count
unassigned
taxon
rank

220638\_out

130

2
superkingdom
130

1239
130
phylum

class
130
186801

130
5

SRS049900\_contig\_number\_contig-100\_6421.120218SRS104975\_contig\_number\_15764SRS1055056\_contig\_number\_contig-100\_3547.80747SRS146812\_contig\_number\_contig-100\_5417.297464SRS893300\_contig\_number\_contig-100\_14905.14906
order
186802

16
family
186806

genus
16
1730


SRS011239\_contig\_number\_23468SRS013158\_contig\_number\_3781SRS971275\_contig\_number\_15310
1263079
3
species


SRS042628\_contig\_number\_38455SRS147766\_contig\_number\_9795
39485
species
2


SRS015217\_contig\_number\_2696SRS019161\_contig\_number\_41481SRS024075\_contig\_number\_contig-100\_4102.97692
142586
3
species

species
8
1897003

SRS011405\_contig\_number\_contig-100\_11112.49215SRS019397\_contig\_number\_9751SRS042284\_contig\_number\_7087SRS047433\_contig\_number\_17596SRS144135\_contig\_number\_contig-100\_4222.195177SRS144506\_contig\_number\_contig-100\_2869.315591SRS147377\_contig\_number\_12456SRS893279\_contig\_number\_17906

543314
family
1


SRS018817\_contig\_number\_1960
1
species
2137877

1
family
31979

genus
1
1485


SRS104311\_contig\_number\_25284
1
species
1262836

107
family
186803


SRS024435\_contig\_number\_contig-100\_1983.249874SRS077194\_contig\_number\_26898
1898203
2
species


SRS011084\_contig\_number\_22596SRS012849\_contig\_number\_32400SRS012969\_contig\_number\_15053SRS013951\_contig\_number\_738SRS014235\_contig\_number\_contig-100\_857.276435SRS014855\_contig\_number\_2274SRS015190\_contig\_number\_4368SRS015217\_contig\_number\_12910SRS015486\_contig\_number\_contig-100\_2643.2644SRS015578\_contig\_number\_contig-100\_2679.205615SRS015579\_contig\_number\_24868SRS015782\_contig\_number\_37027SRS016018\_contig\_number\_1719SRS017103\_contig\_number\_contig-100\_570.238266SRS017191\_contig\_number\_16227SRS017433\_contig\_number\_18003SRS018427\_contig\_number\_32711SRS019601\_contig\_number\_22067SRS019685\_contig\_number\_contig-100\_205.231089SRS020869\_contig\_number\_32374SRS021484\_contig\_number\_31000SRS022137\_contig\_number\_21965SRS022713\_contig\_number\_3058SRS023346\_contig\_number\_7640SRS023526\_contig\_number\_34140SRS023715\_contig\_number\_28120SRS024132\_contig\_number\_36805SRS043001\_contig\_number\_contig-100\_4761.73438SRS043411\_contig\_number\_16381SRS043701\_contig\_number\_contig-100\_509.115152SRS044535\_contig\_number\_24851SRS046717\_contig\_number\_11345SRS047044\_contig\_number\_18522SRS048164\_contig\_number\_31134SRS049446\_contig\_number\_contig-100\_858.99975SRS049712\_contig\_number\_25091SRS049896\_contig\_number\_13671SRS050026\_contig\_number\_contig-100\_3047.75704SRS050925\_contig\_number\_36471SRS050941\_contig\_number\_20427SRS051882\_contig\_number\_contig-100\_1980.138516SRS052697\_contig\_number\_46102SRS053335\_contig\_number\_14978SRS053398\_contig\_number\_contig-100\_5.163139SRS055017\_contig\_number\_35748SRS055982\_contig\_number\_19569SRS056519\_contig\_number\_contig-100\_60.114045SRS057478\_contig\_number\_3108SRS057717\_contig\_number\_4549SRS058770\_contig\_number\_contig-100\_1538.132706SRS063190\_contig\_number\_11570SRS075021\_contig\_number\_8717SRS075821\_contig\_number\_21048SRS075984\_contig\_number\_25092SRS076804\_contig\_number\_20863SRS077231\_contig\_number\_17461SRS077641\_contig\_number\_15240SRS098073\_contig\_number\_18951SRS098571\_contig\_number\_41981SRS098644\_contig\_number\_23284SRS098717\_contig\_number\_22543SRS098881\_contig\_number\_contig-100\_5226.169067SRS103987\_contig\_number\_36816SRS1041036\_contig\_number\_3531SRS1041090\_contig\_number\_7901SRS1041091\_contig\_number\_20948SRS1041133\_contig\_number\_28404SRS1041138\_contig\_number\_contig-100\_9704.9705SRS1041139\_contig\_number\_3478SRS1041144\_contig\_number\_8383SRS1041144\_contig\_number\_contig-100\_9312.9313SRS1041146\_contig\_number\_contig-100\_1069.1070SRS104197\_contig\_number\_contig-100\_966.4016SRS104327\_contig\_number\_7882SRS104636\_contig\_number\_contig-100\_4551.146591SRS105082\_contig\_number\_contig-100\_11070.80424SRS142503\_contig\_number\_12424SRS142890\_contig\_number\_contig-100\_2817.231111SRS143070\_contig\_number\_contig-100\_15.116590SRS143417\_contig\_number\_contig-100\_4883.176004SRS143466\_contig\_number\_28167SRS143598\_contig\_number\_9369SRS143991\_contig\_number\_contig-100\_2107.177423SRS146764\_contig\_number\_contig-100\_2109.198475SRS147271\_contig\_number\_24531SRS147272\_contig\_number\_contig-100\_2229.2230SRS147346\_contig\_number\_24553SRS147614\_contig\_number\_4928SRS147977\_contig\_number\_contig-100\_1086.84731SRS148196\_contig\_number\_40072SRS148253\_contig\_number\_2840SRS148424\_contig\_number\_contig-100\_3620.189242SRS148721\_contig\_number\_33898SRS148817\_contig\_number\_contig-100\_7218.46916SRS149244\_contig\_number\_2576SRS149325\_contig\_number\_contig-100\_1992.112126SRS149784\_contig\_number\_13450SRS893295\_contig\_number\_1940SRS893341\_contig\_number\_15664SRS893342\_contig\_number\_7910SRS893373\_contig\_number\_25151SRS893378\_contig\_number\_contig-100\_42.143060
39491
102
species

3
genus
841
1

SRS149075\_contig\_number\_contig-100\_4039.123268


SRS020233\_contig\_number\_54656
1
species
2293127


SRS019068\_contig\_number\_40204
2049040
species
1
